# CNN-CNN: Dual Convolutional Neural Network Approach for Feature Selection and Attack Detection on Internet of Things Networks

**DOI:** 10.3390/s23146507

**Published:** 2023-07-19

**Authors:** Basim Ahmad Alabsi, Mohammed Anbar, Shaza Dawood Ahmed Rihan

**Affiliations:** 1 Applied College, Najran University, Kind Abdulaziz Street, Najran P.O. Box 1988, Saudi Arabia; baalabsi@nu.edu.sa (B.A.A.); sdrihan@nu.edu.sa (S.D.A.R.); 2 National Advanced IPv6 (NAv6) Centre, Universiti Sains Malaysia, Gelugor 11800, Malaysia

**Keywords:** Internet of Things, IoT attacks, intrusion detection system, feature selection, convolutional neural network

## Abstract

The Internet of Things (IoT) has brought significant advancements that have connected our world more closely than ever before. However, the growing number of connected devices has also increased the vulnerability of IoT networks to several types of attacks. In this paper, we present an approach for detecting attacks on IoT networks using a combination of two convolutional neural networks (CNN-CNN). The first CNN model is leveraged to select the significant features that contribute to IoT attack detection from the raw data on network traffic. The second CNN utilizes the features identified by the first CNN to build a robust detection model that accurately detects IoT attacks. The proposed approach is evaluated using the BoT IoT 2020 dataset. The results reveal that the proposed approach achieves 98.04% detection accuracy, 98.09% precision, 99.85% recall, 98.96% recall, and a 1.93% false positive rate (FPR). Furthermore, the proposed approach is compared with other deep learning algorithms and feature selection methods; the results show that it outperforms these algorithms.

## 1. Introduction

The emergence of the Internet of Things (IoT) as a new paradigm integrating diverse smart devices, sensors, and systems has paved the way for seamless data sharing and automation [1,2]. Many sectors, including healthcare, transportation, smart cities, and manufacturing, will increase the number of IoT devices used by billions by 2030 [3]. The security of IoT devices is an important concern, yet it is not easy to guarantee their proper operation.

IoT network security is complex due to the various devices and protocols employed. IoT networks are more vulnerable to attacks because of the wide variety of connected devices, each of which may have different processing speeds, storage capacities, and security mechanisms. These gadgets’ wireless communication methods are also susceptible to interference and eavesdropping, which might compromise sensitive information and personal privacy [4]. Furthermore, IoT-specific attacks, such as Mirai [5] and Hajime [6], pose significant risks, including damage to reputation and consumer trust. These attacks compromise IoT devices by carrying out various malicious activities, including DDoS attacks. As a result, manufacturers and organizations associated with compromised devices may experience reputational harm, leading to consumer mistrust and hindering IoT adoption. Additionally, IoT-Bot attacks entail financial and legal implications, such as incident response costs and potential legal actions. Therefore, safeguarding reputation and consumer trust is crucial for sustainable IoT growth and acceptance.

Securing IoT networks is further complicated by the inherent instability and distribution of IoT data. Cybercriminals can exploit weaknesses in data flow and conduct attacks, like denial-of-service, man-in-the-middle, and replay attacks, as IoT devices create large volumes of data that must be transferred, stored, and processed swiftly.

Implementing adequate security measures for an IoT infrastructure is difficult enough without the lack of clear rules. The enormous amount of endpoints, the wide range of protocols in use, and the irregularity of traffic make it difficult for traditional intrusion detection systems (IDS) to cope with the intricacies of IoT networks [7].

Researchers have explored various solutions, including conventional cryptography tools, shallow machine learning (ML) algorithms, and deep learning (DL) algorithms, to address the challenges posed by attacks on IoT networks. Among these solutions, convolutional neural networks (CNNs) [8] have emerged as a promising approach to detecting IoT-based attacks. However, further research is needed to investigate the applicability of CNNs in selecting significant features that aid in detecting attacks on IoT networks. Moreover, the performance of CNNs and other DL algorithms relies on selecting appropriate features that serve as patterns for detecting IoT-based attacks. By training DL detection models with suitable features, the performance of these models can be increased.

Therefore, this research aims to explore the potential of utilizing CNNs for feature selection in detecting IoT attacks. While CNNs are commonly used for feature extraction and categorization, their role in selecting the most critical features in IoT-based attacks has not been extensively investigated. By evaluating the effectiveness and suitability of CNNs for this purpose, the research endeavors to contribute to developing reliable and efficient security measures for IoT networks. The main contributions of this research are as follows:

- Leveraging CNNs to select feature sets that help to detect IoT attacks. A CNN’s ability to discover and prioritize crucial features is essential for this attack detection. By applying CNNs to the feature selection process, we aim to enhance the accuracy and efficiency of IoT attack detection systems. Our research contributes to advancing IoT security by demonstrating the effectiveness of CNNs in identifying relevant features for robust attack detection, thereby improving the overall security posture of IoT environments.
- Design and implementation of a CNN-based detection model that utilizes the features selected by the first CNN model. The procedure comprises training the CNN on these features, which ultimately yields a reliable and robust detection system.
- Extensive testing and analysis to evaluate the effectiveness of the proposed approach’s CNN-based feature selection and detection model. This evaluation provides valuable insights into the strengths, limitations, and overall efficacy of the proposed approach in detecting IoT-based attacks.

The remaining sections of the paper are organized as follows: Section 2 provides an overview of IoT security, feature selection, and CNNs. Section 3 reviews related work in attack detection in IoT networks. Section 4 presents the methodology, including using CNNs for feature selection and the design of a detection model. Section 5 presents the results and a discussion of the findings. Section 6 concludes the paper by summarizing the key contributions and suggesting future research directions.

## 2. Background

This section provides an overview of the IoT and its potential vulnerabilities, the potential benefits of CNNs are also provided in this section.

### 2.1. Internet of Things Security

IoT is a system of interconnected gadgets, automobiles, appliances, and other everyday items embedded with electronics, software, and network connectivity to gather and share data [9]. Many sectors and parts of our life stand to benefit greatly from the IoT, yet with this revolutionary potential comes new security threats. The security of IoT devices is typically less than optimal. They may use insecure login credentials or not encrypt or update their data. Because of this, they are susceptible to cyberattacks that may steal information, sabotage systems, or even injure people physically [10,11].

Despite taking precautions to secure IoT networks, the inherent risks associated with IoT networks cannot be completely eliminated. However, regularly updating IoT devices with security patches, implementing data encryption, and maintaining backups are vital practices to ensure ongoing security. By adopting these measures, we can enhance the resilience of IoT networks and safeguard the integrity and privacy of user data [4,7].

IoT-based attacks, such as IoT BoT [12], exploit vulnerabilities in the software and hardware of IoT devices, compromising them to gain access to sensitive information, cause physical damage, or disrupt services. IoT-specific attacks can also be used for network reconnaissance, where attackers probe network infrastructures to gather information about the systems and areas they intend to target. As IoT devices continue to proliferate in our society, the importance of securing these devices against IoT-specific attacks is becoming increasingly important and must be taken into consideration in the design and implementation of IoT technologies.

Achieving comprehensive IoT security requires a multi-layered approach that combines traditional network security measures with specialized security mechanisms. This includes effective device management, strong access controls, secure communication protocols, and safe data storage practices. By adopting these precautions, we can protect sensitive data and prevent unauthorized access to IoT networks, promoting both convenience and security in our lives.

### 2.2. Feature Selection

In machine learning, feature selection narrows a vast feature set to only the most important characteristics. This step is crucial for minimizing the data’s dimensionality, which improves the model’s readability and efficiency. When building a model, careful feature selection may assist in boosting accuracy while cutting down on processing and storage needs [13].

Feature selection approaches may be broken down into three classes: filter, wrapper, and embedding. Statistics are used in filtering procedures to determine which attributes are most important. Wrapper techniques use a model to compare candidate subsets of features and choose the most effective one. During the training phase of a model, embedded approaches involve feature selection, which may boost performance and efficiency [14].

Feature selection is a critical method for identifying IoT threats. In order to detect illegal behavior, it is necessary to choose important attributes from the vast amounts of data produced by IoT devices. Finding the most relevant metrics and attributes that aid in identifying an attack is the goal of this process. Indicative patterns of a cyberattack may be readily identified using machine learning algorithms that have been trained using these attributes [15]. Accurate and efficient detection of IoT-based threats relies heavily on feature selection, which enhances the data quality for training models and speeds up the detection process. Several feature-selection techniques are used to improve the performance of ML and DL algorithms used for detecting IoT-based attacks. Examples of these techniques are principal component analysis (PCA) [16], Autoencoder (AE) [17], the information gain ratio (IGR) [18], chi-square (Chi) [19], and recursive feature elimination (REF) [20]. The criteria for selecting features in IoT threat detection using feature selection techniques are relevancy and discriminability. Relevancy refers to selecting features directly related to the characteristics and behaviors of IoT-based attacks, capturing meaningful information to distinguish between normal and malicious activities in IoT networks. Discriminability means that the selected features should effectively differentiate between different attacks or between attacks and normal network behavior.

However, there are cases when it is not useful or required to pick features. When characteristics are strongly correlated or have a nonlinear connection with the target variable, eliminating them may decrease the model’s accuracy.

### 2.3. Convolutional Neural Networks

Deep neural networks, like CNNs [21], are often employed for a variety of machine learning applications, including natural language processing (NLP), information retrieval (IR), and others. The spatial and temporal patterns of CNNs make them ideal for evaluating and processing visual media and time-series data. In order to learn and extract information from data without human involvement, CNNs employ convolutional layers, which constitute a technical advance. In order to create a feature map, these convolutional layers multiply and sum the input element-wise via a set of trainable filters. The mathematical formula for this is as follows:

(1) $$F\left(i,j\right)=\sigma \left(\sum_{m}\sum_{n}K\left(m,n\right)I\left(i+m,j+n\right)+b\right)$$

In this equation, *F* is the final feature map, *I* is the input data, *K* is the filter set (or kernels), σ is the activation function, *b* is the bias term, and (i,j) are the final feature map’s spatial coordinates. After the features are extracted, they are fed through a sequence of pooling layers to down-sample the output and reduce the data’s dimensionality. Finally, one or more fully connected layers are used for classification or regression tasks.

CNNs can learn and extract features automatically from raw data, doing away with the requirement for feature engineering. This is especially helpful for picture identification and other activities that need computer vision algorithms that are both time-consuming and sophisticated. CNNs are versatile due to their ability to process massive datasets and intricate architectures. They may improve their accuracy and performance by learning to detect more complex patterns and attributes in new data [22]. In the context of IoT, CNNs can be applied in two key roles:

- Feature selection: CNNs can be utilized to select significant features from the dataset that help identify IoT-based attacks. By leveraging their ability to learn intricate patterns and representations from data, CNNs can analyze and prioritize features that are most relevant for detecting IoT attacks. This feature selection process enhances the accuracy and efficiency of IoT threat detection systems.
- Detection model construction: CNNs can be employed to build robust detection models specifically designed to accurately detect IoT-based attacks. These models leverage the learned representations and weights from the CNN layers to effectively analyze and classify IoT data instances as normal or malicious. By training the detection models with appropriate features, CNNs enhance the performance and reliability of IoT attack detection systems.

It is worth mentioning that CNN architectures, like AlexNet, VGGNet, ResNet, and InceptionNet, can be adapted and applied to IoT security scenarios. However, it should be noted that these architectures were primarily designed for image applications, and their adaptation for IoT-based attack detection requires reshaping the input data to a 2D format, which can be resource-intensive and time-consuming. In contrast, 1D CNN architectures are better suited for dealing with tabular data, commonly used for evaluating cybersecurity approaches (e.g., approaches to detect IoT-based attacks). Therefore, for IoT-based attack detection, utilizing 1D CNN architectures that can directly handle tabular data may offer more efficient and effective solutions.

In conclusion, CNNs are a robust neural network type that can learn and extract crucial elements from spatial or temporal input. Since they can automatically learn features from raw data, process enormous datasets, and maintain complicated topologies, they are applicable to various machine learning applications, such as image recognition, NLP, and cybersecurity.

## 3. Related Works

There has been a growing trend toward utilizing deep learning and machine learning algorithms to identify and prevent cyberattacks on IoT networks. Priya et al. [23] proposed a method employing deep neural networks (DNN) to detect attacks on networks, including Internet of Medical Things (IoMT) devices. Their design improved accuracy and reduced computation time by 32% when used in critical cloud computing scenarios, enabling quicker detection and reducing the impact of intrusions. To select the prominent features and achieve anomaly detection in IoT networks in smart cities utilizing deep learning and intrusion detection technologies, Li et al. [24] developed a deep migration learning model architecture. Despite the speed with which their algorithm worked, because of its low detection rate, it was vulnerable to several attacks.

Roopak et al. [25] achieved good accuracy using CNNs with long short-term memory (LSTM) techniques for DDoS attack categorization on the CISIDS-2017 datasets. Hodo et al.  [26] suggested an IDS that uses a multilayer perceptron (MLP) to identify DoS attacks in IoT networks. Mohammed et al. [27] have also recommended IDSs based on Decision Trees (DT), k-Nearest Neighbors (k-NN), and Naive Bayes (NB) for detecting DDoS attacks on IoT devices.

Alimi et al. [28] introduced a revised RLSTM deep learning model to identify DoS attacks in IoT networks. They evaluated the proposed RLSTM model using two standard datasets: CICIDS-2017 and NSL-KDS. The experiments demonstrated that the proposed model substantially enhanced the detection accuracy, precision, recall, and F1 score.

Ge et al. [29] created a novel approach for linked IoT networks that uses deep learning methods. The FFN model of feedforward neural networks was used for binary and multiclass classification. Their program performed well when asked to categorize data into a single category, but it struggled when asked to do so simultaneously. Pecori et al. [30] developed IoT benign and malicious network traces by combining existing data. The dataset had one “normal” category and five different assault types. To obtain the best results, they employed a seven-layer neural network architecture and trained it for 50 iterations. However, the accuracy, recall, and F1 score of the findings for both binary and multiclass classification were less than optimal. Their model proved to be impractical due to its complex structure and poor detection rate.

Susilo and Sari (2020) [31] proposed the use of several machine learning and deep learning strategies, including random forests (RF), CNN, and multi-layer perceptron (MLP), for improving the security performance of IoT networks. The authors developed an algorithm for detecting denial of service (DoS) attacks using a deep-learning algorithm. The BoT-IoT dataset was used to evaluate their work, and it was found that the deep-learning model could increase accuracy, enabling the mitigation of attacks that occur on an IoT network as effectively as possible.

Kaur et al.’s [32] CNN model was utilized to identify and categorize several attacks. The CICIDS-2017 and CICIDS-2018 datasets were utilized to evaluate the effectiveness of their approach. Their model divided attacks into several categories. However, it had poor detection rates for many of them. Ferrag et al. [33] conducted a thorough analysis of deep learning techniques for identifying data security breaches. Seven deep learning models were tested using 35 well-known datasets, and the outcomes were divided into seven categories. On the BoT-IoT [34] and CIC-IDS2018 [35] datasets, the methods for binary and multiclass classification were evaluated, and the results were rated. The effectiveness of various attack tactics against a variety of deep learning models was examined by the authors.

The CNN model developed by Odetola et al. [36] outperformed the feedforward neural network (FFN) and the recurrent neural network (RNN) models in their multilabel classification strategy designed for edge IoT devices. Their method relied heavily on a single convolutional neural network with fixed layers and configurable loss functions. The amount of MAAC processes required was reduced, which also decreased latency. The authors offered a strategy for multilabel identification that would let a model created for standard classification function well in multilabel classification as well. This technique dramatically increased accuracy while saving money in multi-label categorization environments.

## 4. Proposed Approach

**Figure 1:**
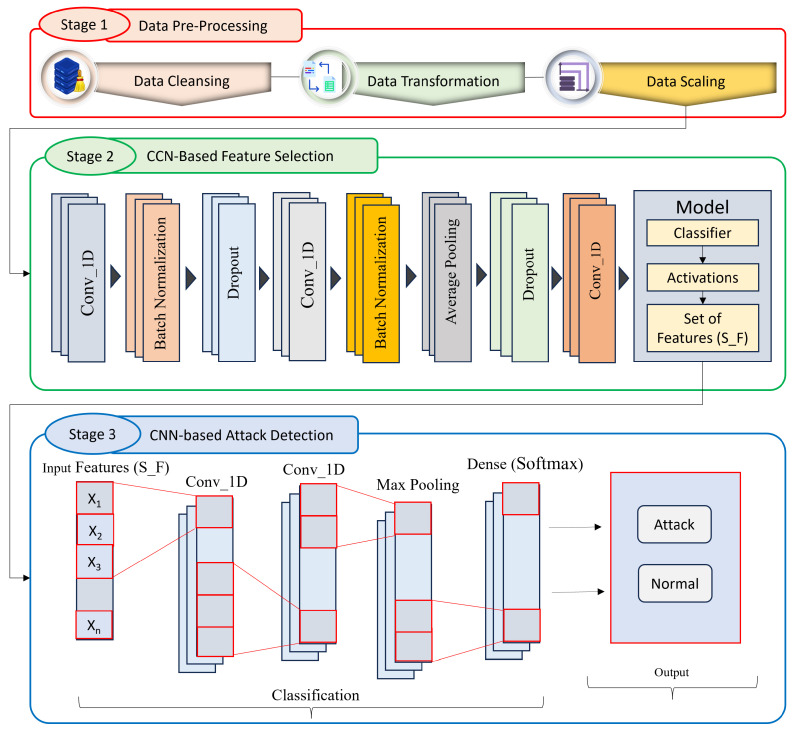
Architecture of the proposed approach.

In this section, we present our proposed approach, CNN-CNN, which utilizes a dual convolutional neural network architecture for feature selection and attack detection on IoT networks. Our approach aims to leverage the power of CNN to automatically select relevant features from IoT network traffic data. CNN-CNN consists of two separate CNN models —one for feature selection and one for attack detection—that work together in a complementary manner to identify the most informative features and accurately classify network traffic as normal or attack. The architecture of the proposed approach is shown in Figure 1.

### 4.1. Data Preprocessing

**Figure 2:**
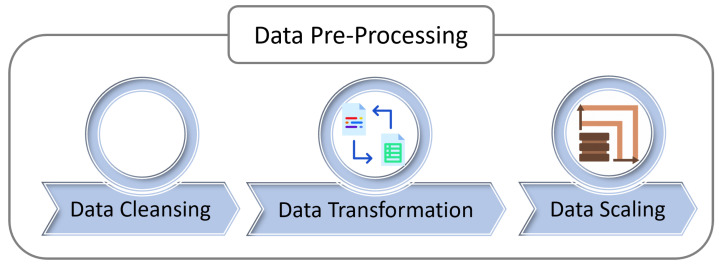
Preprocessing steps.

In the process of preparing data for analysis or modeling, data preprocessing plays a crucial role. This step involves converting raw data into a usable and clean format for machine learning algorithms [37]. To achieve this, several steps are followed in the proposed approach. Firstly, the data is handled to address missing or invalid values, such as infinity or large values, which can affect the quality of the analysis or modeling outcomes. Secondly, removing missing or invalid values is typically the initial step in data preprocessing. Lastly, normalization is often applied to ensure the data is within a defined range. In the proposed approach, a max-min scaler normalization technique [38] is employed, which scales the data between 0 and 1. This technique is particularly useful when data features have different scales or units. Properly preprocessing the data enhances the accuracy of machine learning algorithms in identifying patterns and relationships within the data, which results in better insights and predictions. Figure 2 shows the steps applied to the dataset used.

### 4.2. CNN-Based Feature Selection Stage

This is a core stage of the proposed approach, which aims to utilize 1D CNN for automatically selecting features from raw data. This enhances classification performance and reduces dimensionality. By leveraging the hierarchical and adaptive properties of CNNs, this approach enables precise and efficient feature selection for complex data analysis tasks with high dimensionality. Additionally, using CNNs for feature selection allows us to automatically learn the most informative features from the input data without relying on manual feature engineering. To employ CNN for feature selection, we first train the CNN model using the provided dataset. Then, we retrieve the last convolutional layer from the trained model. Subsequently, we create a new model that takes the same inputs as the original model but outputs the activations of the last convolutional layer. Finally, we calculate the mean activation across all the testing samples, which helps determine the important features. For example, features with high mean activation reflect their importance. The activations of the convolutional layers represent the learned features in the input data, and features with high mean activation indicate their significance. It is worth mentioning that it is possible to compute the mean activations for any convolutional layer in the CNN network, not just the last one. However, we typically concentrate on the last convolutional layer as it contains the most informative and discriminative features contributing to detecting IoT attacks. The architecture of the CNN used for the feature section is tabulated in Table 1.

As shown in Table 1, the last convolutional layer specifically indicates a filter size of 120. This implies that there will be 120 mean activation values generated as output. However, it is important to note that only the values corresponding to the filter indices that are less than or equal to the index of the input data will be considered. Algorithm 1 showcases the pseudocode for CNN-based feature selection.
**Algorithm 1** CNN-based Feature Selection.1:**procedure** CNNFeatureSelection(trained_cnn, f)2:    Create a new model to output the activations of the last convolutional layer3:    Compute the activations for the testing data X4:    Set the desired number of features *n*5:    Find the indices of the features with the highest mean activations6:    and save the output in top_feature_indices variable7:    Initialize an empty list as *top_feature_names* =[]8:    Initialize a counter variable *counter* = 09:    **for each** index **in** *top_feature_indices* **do**10:        **if** index < length(*feature_names*) **then**11:            **if** counter < *n* **then**12:                *top_feature_names* ← *f[index]*13:                *counter*←*counter* + 114:        **else**15:            **continue**16:    **return** *top_feature_names*

The output of this stage is the significant features set $S_{f}$ that is used as the input for the next stage.

### 4.3. CNN-Based IoT Attacks Detection Stage

This stage aims to leverage 1D CNN to generate a robust detection model that can accurately detect IoT attacks, achieving high performance in terms of detection accuracy, precision, and recall. The entry point of this stage is the feature set $S_{f}$ identified in the previous stage. Furthermore, in this stage, the best feature set that demonstrates high CNN performance is selected. This selection is based on metrics such as detection accuracy, precision, and recall. The dataset is divided using the 80/20 rule [39], also known as Pareto theory. Initially, the dataset is stratified to allocate 80% for training and 20% for testing. By employing a stratified approach, we ensure an equal distribution of samples from each category across the training, validation, and testing sets. The stratified methodology is then applied to the training set, dividing it into 80% for training and 20% for validation purposes. The training data is generated based on the selected feature set $S_{f}$ and is utilized to detect IoT attacks in the testing samples. The CNN-based detection model is outlined in Algorithm 2.

Utilizing CNNs as a feature selection technique offers a distinct advantage by surpassing the limitations of filter feature/wrapper selection. This is achieved by harnessing their inherent ability to learn hierarchical representations from input data. Unlike traditional methods, CNNs autonomously extract relevant features without relying on external input or predefined criteria. Consequently, CNNs have the potential to uncover correlations and patterns that may go unnoticed by conventional feature selection techniques. However, the potential limitations of using CNNs for feature selection and attack detection include overfitting, sensitivity to hyperparameters, the need for large amounts of labeled data, and the computational complexity of training deep CNNs. These limitations are not exclusive to CNNs but also apply to other DL algorithms. To address them, researchers and practitioners should validate and evaluate the performance of CNN models for feature selection and attack detection.
**Algorithm 2** CNN-based detection model.1:**procedure** Evaluate1DCNN(X, y, $S_{f}$)2:    **Input:** X (network flows), y (labels), and $S_{f}$ features3:    **Output:** Performance evaluation metrics4:    Define 1D CNN architecture: set activation function, normalization, regularization, and dropout layers5:    Compile 1D CNN model6:    Initialize batch size, optimizer, learning rate, number of epochs (*n*), early stopping criteria (esc)7:    **for** *i* **in** 1 to *n* **do**8:        **while** (esc)9:        Train 1D CNN model using training data based on $S_{f}$ features10:        **end while**11:    Evaluate the 1D CNN model and calculate metrics based on the predictions and *y* labels12:    **Return** the calculated metrics

The proposed approach can be summarized as follows:

- Preprocessing: Preprocess the input data to prepare it for training the CNN. This may include normalization, scaling, and other data-cleaning techniques.
- Build a CNN model: Build a CNN model with multiple convolutional and pooling layers to extract features from the input data. The output of the last convolutional layer can be used as a set of feature maps.
- Train the CNN model: Train the CNN model using the input data to learn the filters that produce the most informative feature maps.
- Compute the mean activations for the last convolutional layer: Extract the feature maps from the testing data using the trained CNN model. Compute the mean activation of each feature map across all instances in the testing dataset.
- Select the most informative features: Identify the most informative feature maps by sorting the mean activations in descending order and selecting the top-k feature maps. These feature maps can be considered the most important features of the given dataset.
- Build a new model using the selected features: Build a new model that uses the selected feature maps as inputs. This new model is used for detecting IoT attacks.

## 5. Experimental Results and Discussion

Through extensive experiments on real-world IoT network traffic datasets, we demonstrate the effectiveness of our approach in achieving high detection rates and low false positive rates, outperforming existing state-of-the-art methods.

### 5.1. Dataset

In this study, we evaluated the performance of our proposed approach using the publicly available dataset IoT-Botnet 2020. This dataset was created by extracting data from PCAP files of the BoT-IoT dataset [34] and is provided in comma-separated values (CSV) format. It includes additional network and flow-based attributes that are relevant to our analysis. The IoT-Botnet 2020 dataset covers a wide range of attack types, including denial of service (DoS), DDoS, reconnaissance, and information theft attacks. This diversity allows us to assess the effectiveness of our approach in detecting various types of IoT-related attacks. Table 2 presents a detailed breakdown of the number of records for the benign and anomaly labels of IoT-Botnet 2020.

As shown in Table 2, the IoT-Botnet 2020 dataset suffers from an imbalance in the label classes, with most cases classified as attacks and a minority as non-attacks. The IoT-Botnet 2020 dataset, despite its class imbalance, is widely used in the research community to evaluate the performance of IoT threat detection methods, such as in [40,41].

In this research, a binary classification approach is employed. Therefore, all instances of attacks present in the BoT-IoT dataset are grouped together under a single category labeled as “attack”.

### 5.2. Evaluation Metrics

The robustness of the proposed approach in detecting IoT attacks is evaluated in terms of several evaluation metrics listed in Table 3.

The metrics listed in Table 3 are considered standard metrics used to evaluate the efficacy of IDS. Furthermore, previous research studies, such as [42,43,44], have employed these metrics to evaluate their own research work.

### 5.3. The Result of CNN-Based Feature Selection Stage

This section aims to present the results of CNN-based feature selection. The CNN architecture, as listed in Table 1, was trained using different batch sizes ranging from 16 to 1024, resulting in the generation of seven feature sets named set1 to set7. Each feature set corresponds to a specific batch size. For example, set1 corresponds to a batch size of 32, set2 corresponds to a batch size of 64, and so on. The diversity of feature sets was achieved by employing various batch sizes, and their impact on the CNN’s performance is discussed in Section 5.4. Before initiating the feature selection process, certain features used in constructing the network flow were excluded from the original feature set. This exclusion was necessary because these features could potentially be used as patterns by the classifier, which could degrade its performance. The eliminated features include Flow_ID, Src_IP, Src_Port, and Dst_IP. Additionally, the features Label, Cat, and Sub_Cat were removed from the original feature set since this research specifically focuses on binary classification, distinguishing between attack and normal instances.

In this research, the top 10 best features were selected from 86 features. This selection was based on sorting the mean activation of test samples in descending order and choosing only the first ten mean activations. Table 4 provides a comprehensive overview of the feature sets based on the corresponding batch sizes.

The output of the stage consists of seven feature sets that contribute to the detection of IoT attacks. These feature sets represent key characteristics and patterns related to IoT network traffic and security threats.

### 5.4. The Result of CNN-Based IoT Attacks Detection

In this stage, CNN is utilized to detect IoT attacks based on feature sets selected in the previous stage. The architecture of the CNN model used to build the detection is the same as shown in Table 1. We employed a sparse categorical cross-entropy loss function in combination with the Adam optimizer to obtain the best possible weight values. In deep learning algorithms, the learning rate is crucial since it determines the size of the model’s steps throughout each iteration. To determine the optimal Adam optimizer learning rate, we conducted a series of trials using rates of 0.01, 0.001, 0.0001, and 0.00001. The 0.01 learning rate is best since it has the highest detection rate. To further lessen the possibility of over-fitting, we used an early preventive technique. If the validation loss has not been reduced after a certain number of training iterations, the training process is terminated. To obtain optimal results during testing, the epoch count should be adjusted such that the network’s accuracy no longer improves. This is the best possible number since the CNN model converged in less than a hundred iterations. Table 5 shows the performance metrics of the CNN for different feature sets (set1 to set7). These metrics are calculated using the equations listed in Table 3.

As shown in Table 5, Set1 achieved a detection accuracy of 95.20%, indicating that it correctly classified 95.20% of instances in the dataset. The precision score was 95.33%, meaning that, out of the instances predicted as attacks, 95.33% were actually true positive detections. The recall score, also known as the sensitivity, was 99.76%, indicating that set1 successfully identified 99.76% of the actual positive instances (IoT attacks). The F1 measure, which balances precision and recall, was 97.49%. The false positive rate (FPR) was 4.69%, implying that 4.69% of the instances predicted as normal were false positives. The AUC-ROC was 64.15%.

Set2 showed higher performance in various metrics. It achieved a detection accuracy of 97.11%, precision of 97.01%, and recall of 99.99%. The F1 measure was 98.48%, indicating a good balance between precision and recall. The FPR was 3.09%, and the AUC-ROC reached 77.45%, indicating better overall performance compared to set1. Similarly, set3, set4, set5, and set6 exhibited varying performance levels regarding detection accuracy, precision, recall, the F1 measure, FPR, and AUC-ROC.

Finally, set7 achieved the highest performance scores among all the feature sets. It achieved a detection accuracy of 98.04% and a precision score of 98.09%, indicating highly accurate detection of IoT attacks. The recall score was 99.85%, indicating the model’s ability to identify a large proportion of actual positive instances. The F1 measure was 98.96%, demonstrating a balanced performance between precision and recall. The FPR was 1.93%, indicating a low rate of false positives. The AUC-ROC was 85.73%, indicating excellent overall performance. In set7 of the IoT-Botnet 2020 dataset, the features can be classified as numerical variables. Bwd_Pkts/b_Avg, Bwd_Seg_Size_Avg, Subflow_Fwd_Byts, Fwd_IAT_Min, Pkt_Len_Std, Bwd_Pkts/s, Fwd_IAT_Tot, Idle_Std, are stored as float64 data type, indicating that they represent continuous numerical values. These features can take on any real number within a certain range. On the other hand, Init_Bwd_Win_Byts and URG_Flag_Cnt are represented as int64 data types, suggesting that they are discrete numerical values.

### 5.5. Discussion

The exceptional performance scores tabulated in Table 5 confirm that a CNN can be leveraged to identify significant features that contribute to detecting IoT attacks. Additionally, Table 5 reveals that set7 comprises the most significant features that contribute to the accurate detection of IoT attacks. Furthermore, the impact of set7 is evaluated on various other deep learning models used in existing research works to assess their ability to accurately detect IoT attacks, including long short-term memory (LSTM) [45], recurrent neural network (RNN) [46], and gated recurrent units (GRUs) [47]. The architecture of LSTM, RNN, and GRUs are based on [48]. Table 6 shows the performance results of LSTM, RNN, and GRUs based on set7.

**Figure 3:**
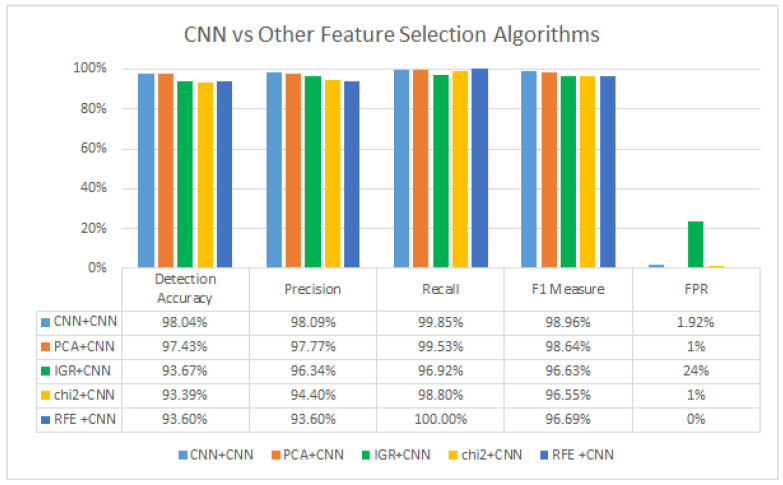
CNN vs. Other Feature Selection Algorithms.

Table 6 demonstrates that the CNN model generally performs better in terms of accuracy, precision, and AUC-ROC compared to the other models. Finally, the CNN is compared with other feature selection techniques, namely PCA [16], AE [17], IGR [18], Chi [19], and REF [20]. The default parameters were employed for the feature selection techniques used in this study. The REF technique utilized a logistic regression estimator. Table 7 presents the top 10 features selected by each algorithm. The impact of these selected features on the performance of the CNN is illustrated in Figure 3.

The “CNN+CNN” model demonstrates the highest overall performance, achieving a detection accuracy of 98.04%. This indicates that the model is highly effective in correctly identifying IoT attacks. The precision of 98.09% suggests that the model has a low false positive rate, meaning it accurately identifies true positive instances. The recall rate of 99.85% indicates a high true positive rate, suggesting that the model effectively captures a large proportion of actual positive instances. The F1 measure, which combines precision and recall, is 98.96%, indicating a balance between precision and recall. The FPR of 1.92% is relatively low, suggesting that the model has a good ability to avoid misclassifying negative instances as positive. Using CNNs as the feature selection technique overcomes the limitations of filter feature/wrapper selection by leveraging their inherent capacity to learn hierarchical representations from the input. Unlike traditional approaches, CNNs autonomously extract relevant features from data without external input or predefined criteria. In this way, CNNs may find correlations and patterns that would have been missed by traditional feature selection techniques. By harnessing their adaptive learning capability, CNNs provide a more effective approach to feature selection in tackling the challenges of IoT threat detection.

In summary, the results demonstrate that the “CNN+CNN” model outperforms others in accuracy, precision, recall, and F1 measure, confirming the effectiveness of leveraging a CNN for feature selection and the detection process. Notably, the “PCA+CNN” model stands out with its remarkable performance, exhibiting an exceptionally low false positive rate. The “IGR+CNN”, “chi2+CNN”, and “RFE+CNN” models also demonstrate respectable performance, albeit slightly lower than the top-performing “CNN+CNN” model. These findings highlight the importance of selecting appropriate feature selection algorithms in conjunction with a CNN to optimize the performance of IoT attack detection systems.

## 6. Conclusions and Future Work

This paper presents an approach for detecting attacks on IoT networks using a combination of two convolutional neural networks (CNN-CNN) for feature selection and building a robust detection model. By training two independent CNNs, our approach effectively detects the IoT attacks in IoT networks. The second CNN leverages the features identified by the first CNN, which are derived from raw network traffic data, to detect attacks.The proposed approach overcomes the limitations of traditional feature selection techniques by autonomously learning hierarchical representations from input data, uncovering correlations and patterns missed by other methods. This adaptive learning capability makes CNNs more effective for feature selection in IoT threat detection. Evaluation of our proposed approach on a publicly BoT-IoT 2020 dataset demonstrated its high performance. We achieved an impressive accuracy rate of 98.04% and 1.93% in terms of detection accuracy and false positive rate, respectively. These results highlight the effectiveness and robustness of our approach in detecting a wide range of threats in IoT networks. For future work, several directions can be explored further to enhance the performance and applicability of our approach. Firstly, incorporating additional layers or architectures into the CNN models may help capture more complex patterns and improve detection capabilities. Secondly, exploring different feature selection techniques or leveraging other machine learning algorithms in conjunction with CNNs could potentially enhance the overall detection accuracy. The proposed future directions offer potential benefits, such as improved detection capabilities through the integration of additional layers or architectures and autonomous feature selection without external input. However, these directions also pose challenges, including increased complexity and resource requirements, effective generalization and scalability, hyperparameter tuning, and thorough evaluation and validation to ensure practical applicability in IoT threat detection. Finally, it would be beneficial to explore mathematical models and logic to verify the correctness of the proposed approach. By developing rigorous mathematical models and logical frameworks, researchers can ensure that the approach is sound, reliable, and effective in detecting IoT-based attacks.

## Figures and Tables

**Table 1:** Architecture of the CNN used for feature section.

Layer (Type)	Output Shape	Number of Parameters	Layer/Technique Sequence
conv1d_4 (Conv1D)	(None, 79, 4)	16	1
batch_normalization_6	(None, 79, 4)	16	2
dropout_6	(None, 79, 4)	0	3
conv1d_5 (Conv1D)	(None, 75, 16)	336	4
batch_normalization_7	(None, 75, 16)	64	5
average_pooling1d_2	(None, 37, 16)	0	6
dropout_7	(None, 37, 16)	0	7
cnov1 (Conv1D)	(None, 33, 120)	9720	7
batch_normalization_8	(None, 33, 120)	480	8
dropout_8	(None, 33, 120)	0	9
flatten_2	(None, 3960)	0	10
dense_2 (Dense)	(None, 2)	7922	11

**Table 2:** The row distribution of IoT-Botnet 2020.

No. of normal category records	40,073
No. of attack category records	585,710
Total no. of records	625,783
No. of features	85

**Table 3:** Evaluation metrics.

Evaluation Metric	Definition
Precision	The ratio of accurately predicted attacks to all samples predicted as attacks. Precision = TP/(TP + FP)
Recall or True Positive Rate (TPR)	The proportion of all attack samples correctly classified as attacks vs. all attack samples. Recall = TP/(TP + FN)
False Positive Rate (FPR)	The ratio of incorrectly predicted attack samples vs. all normal samples. False Alarm Rate = FP/(TN + FP)
Accuracy	The proportion of instances correctly classified vs. the total number of instances. Accuracy = (TP + TN)/(TP + TN + FP + FN)
F1 measure	The harmonic means of precision and recall. F1 Measure = 2 × (Precision × Recall)/(Precision + Recall)
AUC-ROC	The AUC-ROC is computed by plotting the TPR against the FPR at various classification thresholds and calculating the area under this curve. AUC-ROC=∫TPR(FPR)dFPR

**Table 4:** Feature sets based on batch size.

Batch Size	Selected Features
16	Fwd_IAT_Min, Flow_IAT_Mean, TotLen_Bwd_Pkts, Bwd_Pkt_Len_Std, Fwd_IAT_Tot, Fwd_Pkt_Len_Max, Pkt_Size_Avg, Bwd_Pkts/s, Flow_Pkts/s, Bwd_IAT_Std
32	Flow_IAT_Max, Bwd_Header_Len, Flow_Pkts/s, Fwd_Pkts/s, Idle_Max, Bwd_Pkt_Len_Min, Pkt_Len_Std, SYN_Flag_Cnt, Bwd_IAT_Min, Active_Mean
64	Fwd_Seg_Size_Avg, RST_Flag_Cnt, Fwd_Seg_Size_Min, ECE_Flag_Cnt, ACK_Flag_Cnt, URG_Flag_Cnt, Fwd_Byts/b_Avg, Pkt_Size_Avg, Bwd_Header_Len, Fwd_IAT_Std
128	Fwd_URG_Flags, Bwd_Blk_Rate_Avg, Init_Bwd_Win_Byts, Flow_IAT_Std, Subflow_Bwd_Pkts, Down/Up_Ratio, Bwd_PSH_Flags, Fwd_Blk_Rate_Avg, Bwd_Pkt_Len_Min, Flow_Byts/s
256	Idle_Std, Flow_IAT_Min, RST_Flag_Cnt, Tot_Fwd_Pkts, URG_Flag_Cnt, Fwd_Seg_Size_Min, Subflow_Bwd_Byts, CWE_Flag_Count, Idle_Max, Flow_Duration
512	Fwd_Act_Data_Pkts, Idle_Std, Bwd_Pkt_Len_Min, CWE_Flag_Count, Bwd_Pkt_Len_Max, Fwd_Pkt_Len_Min, Pkt_Len_Min, Fwd_IAT_Min, Fwd_Blk_Rate_Avg, RST_Flag_Cnt
1024	Bwd_Pkts/b_Avg, Bwd_Seg_Size_Avg, Subflow_Fwd_Byts, Fwd_IAT_Min, Pkt_Len_Std, Bwd_Pkts/s, Fwd_IAT_Tot, Init_Bwd_Win_Byts, Idle_Std, URG_Flag_Cnt

**Table 5:** Performance Metrics of CNN for Different Feature Sets.

Feature Set	Detection Accuracy	Precision	Recall Score	F1 Measure	FPR	AUC-ROC
Set1	95.20%	95.33%	99.76%	97.49%	4.69%	64.15%
Set2	97.11%	97.01%	99.99%	98.48%	3.09%	77.45%
Set3	95.00%	95.41%	99.45%	97.39%	4.68%	64.75%
Set4	96.99%	97.06%	99.81%	98.41%	2.96%	77.78%
Set5	97.39%	97.54%	99.73%	98.62%	2.46%	81.48%
Set6	95.96%	96.08%	99.74%	97.88%	3.98%	70.17%
Set7	98.04%	98.09%	99.85%	98.96%	1.93%	85.73%

**Table 6:** Performance results of LSTM, RNN, and GRUs based on set7.

Model	Detection Accuracy	Precision	Recall Score	F1 Measure	FPR	AUC-ROC
CNN	98.04%	98.09%	99.85%	98.96%	1.92%	85.73%
RNN	96.44%	96.72%	99.57%	98.12%	3.29%	75.08%
LSTM	96.54%	96.68%	99.73%	98.18%	3.32%	74.87%
GRU	96.60%	96.92%	99.53%	98.21%	3.07%	76.68%

**Table 7:** Best Top 10 selected feature by each algorithm.

Algorithm	Selected Features
CNN	[‘Bwd_Pkts/b_Avg’, ‘Bwd_Seg_Size_Avg’, ‘Subflow_Fwd_Byts’, ‘Fwd_IAT_Min’, ‘Pkt_Len_Std’, ‘Bwd_Pkts/s’, ‘Fwd_IAT_Tot’, ‘Init_Bwd_Win_Byts’, ‘Idle_Std’, ‘URG_Flag_Cnt’]
PCA	[‘Flow_Duration’, ‘Tot_Fwd_Pkts’, ‘Tot_Bwd_Pkts’, ‘TotLen_Fwd_Pkts’, ‘TotLen_Bwd_Pkts’, ‘Fwd_Pkt_Len_Max’, ‘Fwd_Pkt_Len_Min’, ‘Fwd_Pkt_Len_Mean’, ‘Fwd_Pkt_Len_Std’, ‘Bwd_Pkt_Len_Max’]
AE	[‘Bwd_Pkt_Len_Max’, ‘Tot_Bwd_Pkts’, ‘Fwd_Pkt_Len_Mean’, ‘Fwd_Pkt_Len_Max’, ‘Tot_Fwd_Pkts’, ‘TotLen_Fwd_Pkts’, ‘TotLen_Bwd_Pkts’, ‘Fwd_Pkt_Len_Min’, ’Flow_Duration’, ‘Fwd_Pkt_Len_Std’]
IGR	[‘Flow_Duration’, ‘TotLen_Bwd_Pkts’, ‘Flow_Byts/s’, ‘Flow_IAT_Mean’, ‘Bwd_Header_Len’, ‘RST_Flag_Cnt’, ‘Subflow_Fwd_Byts’, ‘Subflow_Bwd_Byts’, ‘Active_Max’, ‘Idle_Mean’]
chi2	[‘Fwd_Pkt_Len_Max’, ‘Fwd_Pkt_Len_Mean’, ‘Fwd_Pkt_Len_Std’, ‘Bwd_Pkt_Len_Min’, ‘Pkt_Len_Max’, ‘Pkt_Len_Mean’, ‘Pkt_Len_Var’, ‘RST_Flag_Cnt’, ‘Down/Up_Ratio’, ‘Subflow_Bwd_Byts’]
REF	[‘Flow_Duration’, ‘Fwd_Pkt_Len_Std’, ‘Flow_Byts/s’, ‘Flow_IAT_Mean’,‘Bwd_URG_Flags’, ‘Bwd_Header_Len’, ‘Bwd_Pkts/s’, ‘RST_Flag_Cnt’, ‘Subflow_Bwd_Byts’, ‘Idle_Mean’]

## Data Availability

Not applicable.
